# Are You Still a Postdoc? How My Scientific Identity Intersects with My Immigrant Status

**DOI:** 10.1128/mSphere.00372-20

**Published:** 2020-05-06

**Authors:** Emma Hernandez-Sanabria

**Affiliations:** a VIB, Center for Microbiology, KU Leuven Laboratory of Molecular Bacteriology, Rega Institute, Leuven, Belgium

**Keywords:** academia, diversity, immigrant, postdoctoral fellow

## Abstract

Academics in non-tenure-track positions encounter a unique set of challenges on the road toward tenure. Institutionalized policies and lack of mentors are additional burdens for foreign scientists, resulting in representation differences. Becoming a scientist has been a personal and moving journey in which my multiple selves intersect and clash every now and again. My identity as a scientist is a life project and has intersected with my other identities: a young Latina immigrant in Western Europe. This crossroad has molded, and at times, challenged my participation in science.

## Abstract (precis)

Academics in non-tenure-track positions encounter a unique set of challenges on the road toward tenure. Institutionalized policies and lack of mentors are additional burdens for foreign scientists, resulting in representation differences. Becoming a scientist has been a personal and moving journey in which my multiple selves intersect and clash every now and again. My identity as a scientist is a life project and has intersected with my other identities: a young Latina immigrant in Western Europe.

## COMMENTARY

My career has many commonalities with those of other young researchers. I have enjoyed science since my early years; I was good at it, and being in Europe has given me the opportunity to travel, experience multiculturalism, and expand my imagination. Beyond that, science provided me with a personal identity and the sense of being part of a community. However, navigating visa and immigration issues is a task that can be overlooked by academic and research institutions. Upon completing my bachelor’s degree in Mexico, I was accepted into a master’s program in the United Kingdom. On arrival, the customs officer at Aberdeen International Airport was unconvinced by the proofs of funding for my studies. My passport was retained, and I was told that I was going to be deported 2 days later. Fortunately, the welcoming team from the university picked me up at the airport. A staff member provided support and assisted me in successfully refuting the resolution of the officer. I often wonder what I would have done without any advice on the spot. The apology letter from the Immigration Office vanished during one of my travels, but the mark on my passport remained until I changed my document.

Following a fulfilling period of doctoral studies in Canada, my passion for research led me to start a new journey in Europe. I have been a postdoctoral fellow for 6 years in the European Union (EU), and the time spent in the same position has raised questions about my future, prospects, and goals. The uncertainty of working in research while an immigrant oftentimes restricts the creation of expectations for settling down (1). As a Mexican citizen, my visa had to be renewed each year, even with a 3-year contract. If I wanted to make long-term plans, it was suggested that I find a permanent position. I worked relentlessly to cement the identity of a competent scientist, confident that my abilities would allow me to conquer that permanent spot. Women are in the minority among senior academics in Europe (15%) (2), although by 2030, our participation in science, technology, engineering, and mathematics (STEM)-related fields will increase to contribute between 0.7 and 0.9 the gross domestic product (GDP) per capita of the EU (3). Europe now needs 1 million additional researchers in STEM (4), so I relied on my institutions to back my scientific identity and to support me in achieving tenure. Career recruitment, progression, and the organizational culture of European academia are influenced mostly by informal interactions, and local conditions regulate integration into the system (5, 6). Joining and participating in the multiple academic spheres require adopting the local language, making it harder for a newcomer to immediately fit in. As job security was uncertain, I assertively and proactively attempted to pursue opportunities to stay in the system and eventually change immigration status. The initial stresses associated with understanding a different institutional culture and balancing work and a private life compounded the instability of my migration status, turning my journey into a struggle. My contract ended, and the paperwork for starting a new postdoc was submitted, but no formal contract was generated. The local immigration office advised me to leave the Schengen Area to avoid remaining illegally, and I had only a few days to organize my departure from the country where I had been for 3 years. I left behind my partner, apartment, and possessions, expecting that the customs officer back home would let me reenter the EU as a tourist 1 week later.

I love my work, and I offered my loyalty and time because I think that from my position in academia, I can inspire and help others to discover their strengths and to realize their potential. I saw the changes that I made through my service. Mentoring gave the opportunity of building up the confidence and identities of younger researchers. Comments from mentees, such as “the passion with which Emma talked about the subject boosted my motivation, even when my own enthusiasm was at a low tide” and “you were a source of knowledge, motivation and support but above all a source of inspiration,” fueled my own motivation. Contrarily to the principles of meritocracy, the different dimensions of my academic work and my contribution to the public good were not valued and did not lead to stability. Providing appreciation is not an inordinate request but an approach that principal investigators can use to give back to society and spark social change. External recognition often enables internal recognition. Indeed, advocating for mentees and boosting their visibility can help minorities to achieve leading positions (7), while ethnic diversity supports scientific impact (8). Hence, accountability from universities and funding agencies is key for achieving fair and diverse representation at higher levels of European STEM.

Perhaps the lack of recognition and the absence of Latina role models in Europe influences the faculty’s perspective of us as prospective elements of the community. Awareness of the privileges that I accessed as a result of my earlier education and my experiences as an immigrant prompted me to act as an example of a minority (immigrant/Latina researchers) that needs visibility and that is frequently stereotyped as lazy, overly emotional, and superficial (9). Minorities have traditionally resorted to develop a “thick skin,” reinventing career paths and paving the way for new generations. Female, immigrant, Latina, and underrepresented mentors can become crucial gatekeepers to resources, networking, and support. If it takes a village to raise a child, why does a village not stick together when one is taking baby steps toward fulfilling career goals? We need the assistance of the players involved in securing our future in our career of choice. I have enjoyed and benefitted from collaborative and interdisciplinary research, and I see myself as consistent, rather than stagnant, but the lack of recognition and structural support for career advancement (6) impacted my overall well-being. Addressing the mental health crisis of early-career researchers calls for urgent action (10, 11). Being perceived as vocal, emotional, or enthusiastic should not lead to negative recognition anymore. Inclusive leadership styles have some of these components. Language and physical appearance are part of our immigrant and social identities and can align with being a STEM professional. We do not want to take the place of or be someone else. We want the opportunity to be ourselves and use our distinctive backgrounds and capabilities to benefit STEM in achieving their inclusive and scientific objectives.
